# Neo4j graph database realizes efficient storage performance of oilfield ontology

**DOI:** 10.1371/journal.pone.0207595

**Published:** 2018-11-16

**Authors:** Faming Gong, Yuhui Ma, Wenjuan Gong, Xiaoran Li, Chantao Li, Xiangbing Yuan

**Affiliations:** 1 Department of Computer Technology, College of Computer and Communication Engineering, China University of Petroleum, Qingdao, Shandong, China; 2 China Petroleum and Chemical Corporation Shengli Oilfield Branch Ocean Oil Production Plant, Dongying, Shandong, China; Polytechnical Universidad de Madrid, SPAIN

## Abstract

The integration of oilfield multidisciplinary ontology is increasingly important for the growth of the Semantic Web. However, current methods encounter performance bottlenecks either in storing data and searching for information when processing large amounts of data. To overcome these challenges, we propose a domain-ontology process based on the Neo4j graph database. In this paper, we focus on data storage and information retrieval of oilfield ontology. We have designed mapping rules from ontology files to regulate the Neo4j database, which can greatly reduce the required storage space. A two-tier index architecture, including object and triad indexing, is used to keep loading times low and match with different patterns for accurate retrieval. Therefore, we propose a retrieval method based on this architecture. Based on our evaluation, the retrieval method can save 13.04% of the storage space and improve retrieval efficiency by more than 30 times compared with the methods of relational databases.

## Introduction

The ontology is the formal representation of knowledge as a set of concepts and the relationships within a domain [1]. As an important part of knowledge engineering, domain ontology describes the concept of a specific discipline [2]. The domain ontology generally has four parts: domain disciplines, concept attributes, concept attributes, and attribute relationship constraints. All these parts can demonstrate specific knowledge in a domain, which typically includes Resource Description Framework (RDF), Web Ontology Language (OWL), or other terms [3]. The oilfield ontology describes various concepts of knowledge in the petroleum industry, as well as the interrelationships between concepts, domain activities, and domain characteristics. According to the oilfield ontology, multidisciplinary knowledge integration and information integration in the oilfield illustrated the relationship between terminology and their domain axiom in order to formally describe them. With the deepening of oil exploitation and the rapid expansion of big data, more and more petroleum industries have chosen domain ontology for knowledge management. Even though significant progress has been achieved in ontology data management, storage and search are costly and demanding [4]. Many domain ontology datasets are quite large, which brings great challenges to the establishment of the library.

The storage of relational databases adopts organizational technology and can be divided into three categories according to their storage structure: triple-table, horizontal partition and vertical partition. A triple-table stores all the RDF data in a single three-column table, where each row is an RDF statement. Although the triple-table method has superior performance for small-scale data, as the size of the data increases, it will generate a large number of self-joins. The horizontal partitioning represents conceptually stores all the RDF data in a single table, where the columns store each predicate value of the RDF graph. Although this wide-table method supports multi-valued attributes, the storage method is not suitable for large-scale data storage because sparse attributes result in a large number of empty cells. The vertical partitioning rewrites the triple-table into *n* two-column tables, where *n* is the number of unique properties in the data. It is easily implemented and performs well for queries that specify the predicate values but is otherwise not a good approach, because the retrieval time of information increases exponentially with the volume of data.

**Fig 1:**
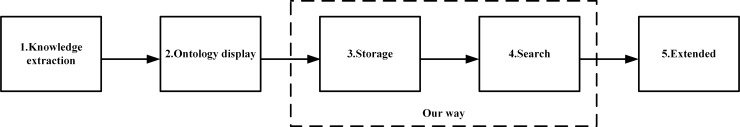
Ontology construction flow chart.

The ontology construction method [5–7] can be divided into manual construction and semi-automatic construction. Researchers have proposed many construction methods, such as Skeletal Methodology, IDEF-5, Methontology, TOVE enterprise modeling and cyclic acquisition. It (Fig 1) depicts an ontology construction process inspired by the classical skeletal methodology (SM).

However, in addition to the SM approach, we also considered the multidisciplinary nature of the petroleum sector and the need for later ontology extensions, so we developed specific processes. The steps are as follows.

1. Knowledge extraction is the acquisition of knowledge in ontology. The expert extracts knowledge from the relevant field and establishes a link for a relevant concept.
2. The Web Ontology Language uses logical representations rather than natural language to represent the ontology construction process.
3. A suitable storage program can improve the query and management efficiency of the ontology.
4. The user can use the ontology to search for and acquire needed knowledge.
5. The extension is the process of adding new ontology information or integrating different ontologies based on the original ontology.

In this paper, we focus particularly on storage and search. We devise Neo4j database mapping rules for ontology files (RDF data). A mapping relation is established through the data structure of the RDF graph and the storage structure of the Neo4j database, and the effective dump of ontology data in Neo4j is realized. At the same time, we adopt a two-tier index architecture, including object and triad indices, to keep loading times low and match with different patterns for accurate retrieval. Thus, we propose a retrieval method based on this architecture. According to our evaluation, the retrieval method can save 13.04% less storage space and improve retrieval efficiency by more than 30 times compared to relational database methods.

The remainder of this paper is organized as follows. In section 2, we provide an overview of related work on RDF data storage and retrieval in the Neo4j database. Section 3 describes the mapping relationship between RDF data and the Neo4j data model, and presents our data-storage and query-processing mechanisms. The experimental evaluation of our approach is described in section 4. Section 5 provides our conclusions and identifies future research directions.

## Related work

The appropriate ontology storage method is conducive to the extension of the ontology and the improvement of query efficiency. Many people use relational databases in ontology storage. Loan [8] proposed an approach of transforming ontologies into relational databases, which presents the principles of mapping OWL concepts to relational database schemas is presented with an implemented tool. Since relational database data are stored in a two-dimensional table, and the ontology model uses the map structure of directed graph, an impedance-mismatch problem will occur in the conversion process [9]. Elbauah[10] selected an object-oriented database for ontology storage. Although the ontology-storage model based on an object-oriented database is suitable for data-storage of complex relationships, this technology is still insufficient to support a massive oil domain ontology database in a big-data environment.

In order to solve the above problems, Rani [11] proposed an ontology-driven system to verify the integration of ontology and semantic Web environments by implementing the Felder-Silverman learning style model. Vysniauskas [12] focuses on the characteristics of OWL ontology and attributes. They improve the existing schema by setting the relational table and adding the relation constraint table Trend to make it easier to implement the information storage of classes, attributes and complex relationships in the OWL ontology. In addition, domain ontology storage has a huge impact on gene ontology in bioinformatics. Dietze [13] proposed biological ontologies such as the gene ontology (GO) and the human phenotype ontology (HP) that provided a rich set of constructs for describing biological entities such as genes, alleles and diseases. Overton et al [14] proposed to use XOD strategy and powerful XOD tool development to greatly support ontology interoperability and powerful ontology applications to support searchable, accessible, interoperable and reusable data. With the continuous improvement of the information level in the petroleum field, although the ontology storage can be applied to many fields, the rapid growth of data volume will bring more problems to the ontology storage and retrieval.

The RDF describes specific information about various applications on the World Wide Web [15]. An RDF uses the three-tuple form of the subject, predicate, and object to describe the resources on the Web. The subject generally uses the Uniform Resource Identifier (URI) to represent the information entity (concept) on the Web, the predicate description contains the relevant attribute, and the object is the corresponding attribute value. This expression of an RDF can be used to represent any identified information on the web and makes it possible to exchange RDFs between applications without loss of semantic information. Hence, the RDF has become the standard for semantic data description, which is widely used in metadata description, domain ontology and the Semantic Web.

**Fig 2:**
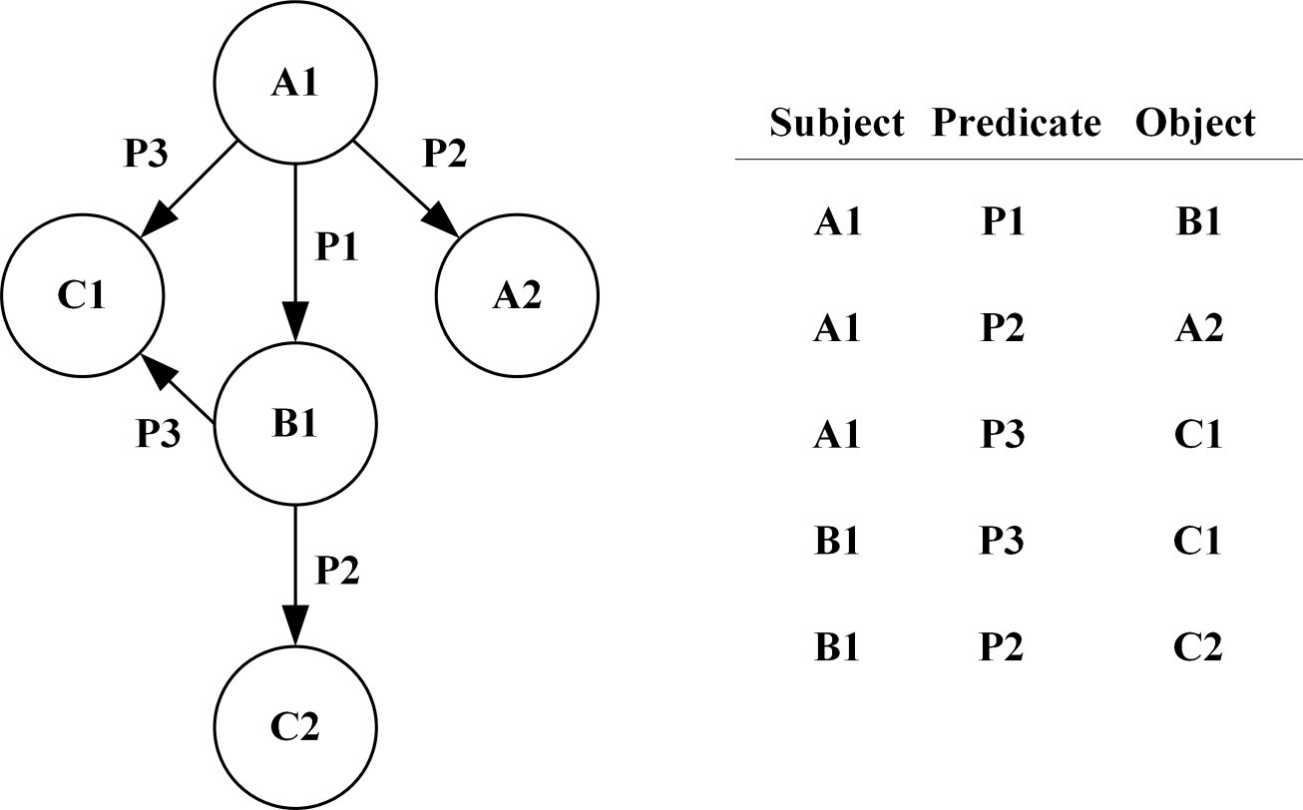
An RDF graph and corresponding triples relationship.

The RDF data is represented as (*S*,*P*,*O*) triples [16]:

T=〈s,p,o〉∈(I∪B)×I×(I×B×L)

where T is the set of RDF triples, I denotes uniform resource identifiers (IRIs), B denotes empty nodes, and L denotes literals. An RDF directed graph can be represented by a labeled node and a labeled edge, and it states the relationship between the subject and the object to which it refers. An RDF directed graph is the subject of all the triples it contains, and the direction of the edge always points to the object. A set of RDF data can usually form an RDF directed graph. Fig 2 shows an RDF chart and the triad table it contains.

The oil exploration and development field includes more than 20 branch fields such as geology, exploration, drilling, machinery, underground operations, oil production and oil storage. The domain ontology is described by OWL-DL language and stored by OWL files. The ontology data can be represented RDF triples by parsing OWL files, and multiple RDF triples can form an RDF directed graph. Storage of the ontology data is the storage of RDF directed graph data, and the storage of RDF directed graph data is essentially the storage of many RDF triples.

**Fig 3:**
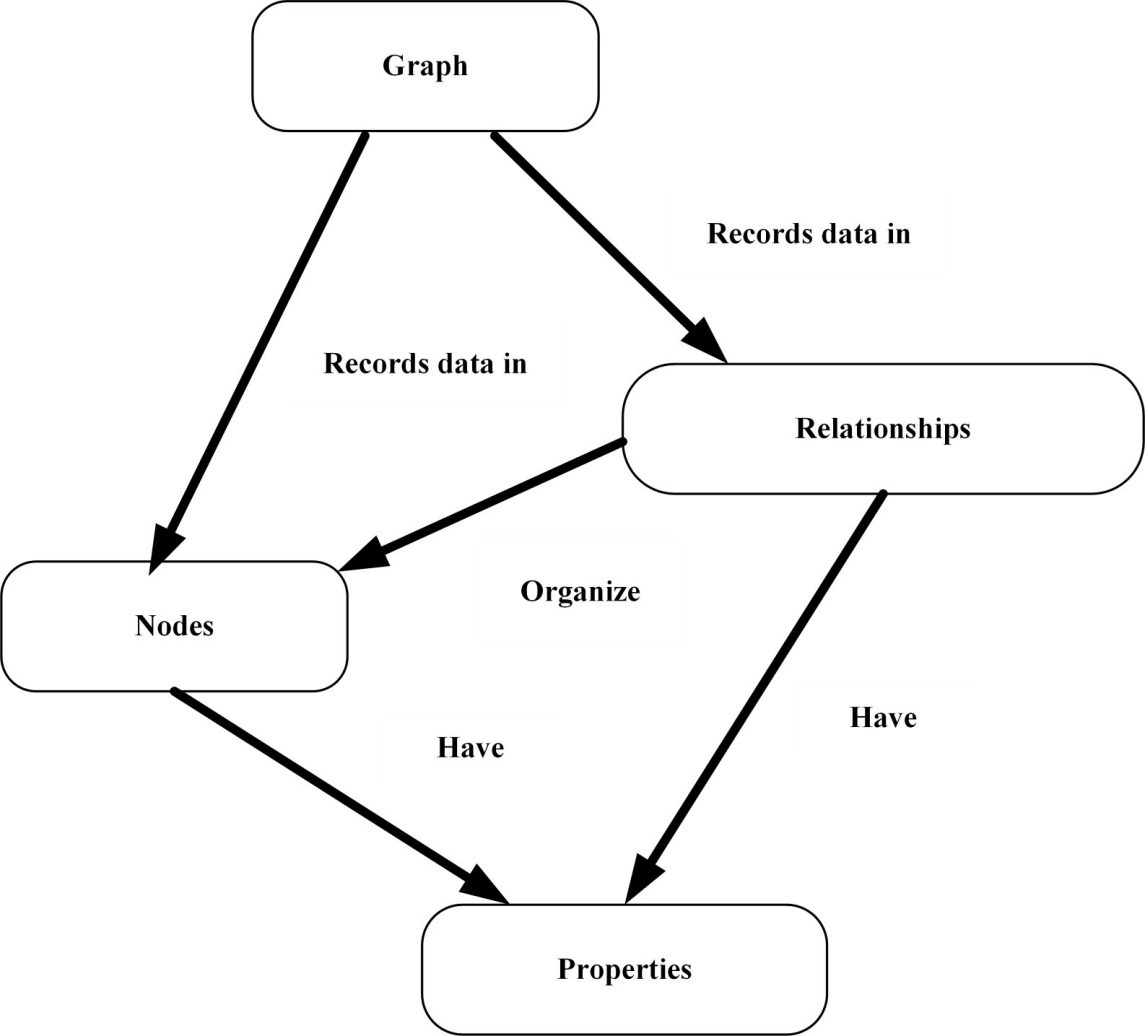
The data structure of Neo4j.

Neo4j uses graph structure as its storage structure, which is a general data structure that can model data and give it powerful expressive power. Linked list, tree, and hash tables and other data structures can be expressed by an abstract network. Neo4j has the characteristics of the attribute graph data model, which can flexibly expand its network model. Its primitive consists of three elements: node, relationship and attribute, which can completely describe the situation of many users. The advantage of this storage model is that the node attributes of the storage model can be added or deleted at any time, effectively solving the problem of semi-structured, unstructured data storage and memory waste. In addition, due to its unique data model, the Neo4j database can quickly query information node related information through deep traversal and other methods. Fig 3 shows the data structure of Neo4j.

## Our method

This paper uses Protégé software and the classic skeleton method (SM) to construct the oilfield ontology data [17]. Protégé software is an open source ontology editing and knowledge acquisition software developed in the Java language. The OWL is used to build RDF datasets in the oilfield. It combines hybrid reasoning with the Jayton inference engine to add rich semantic information by adding blending rules.

### 3.1 Structures of oilfield ontology

**Fig 4:**
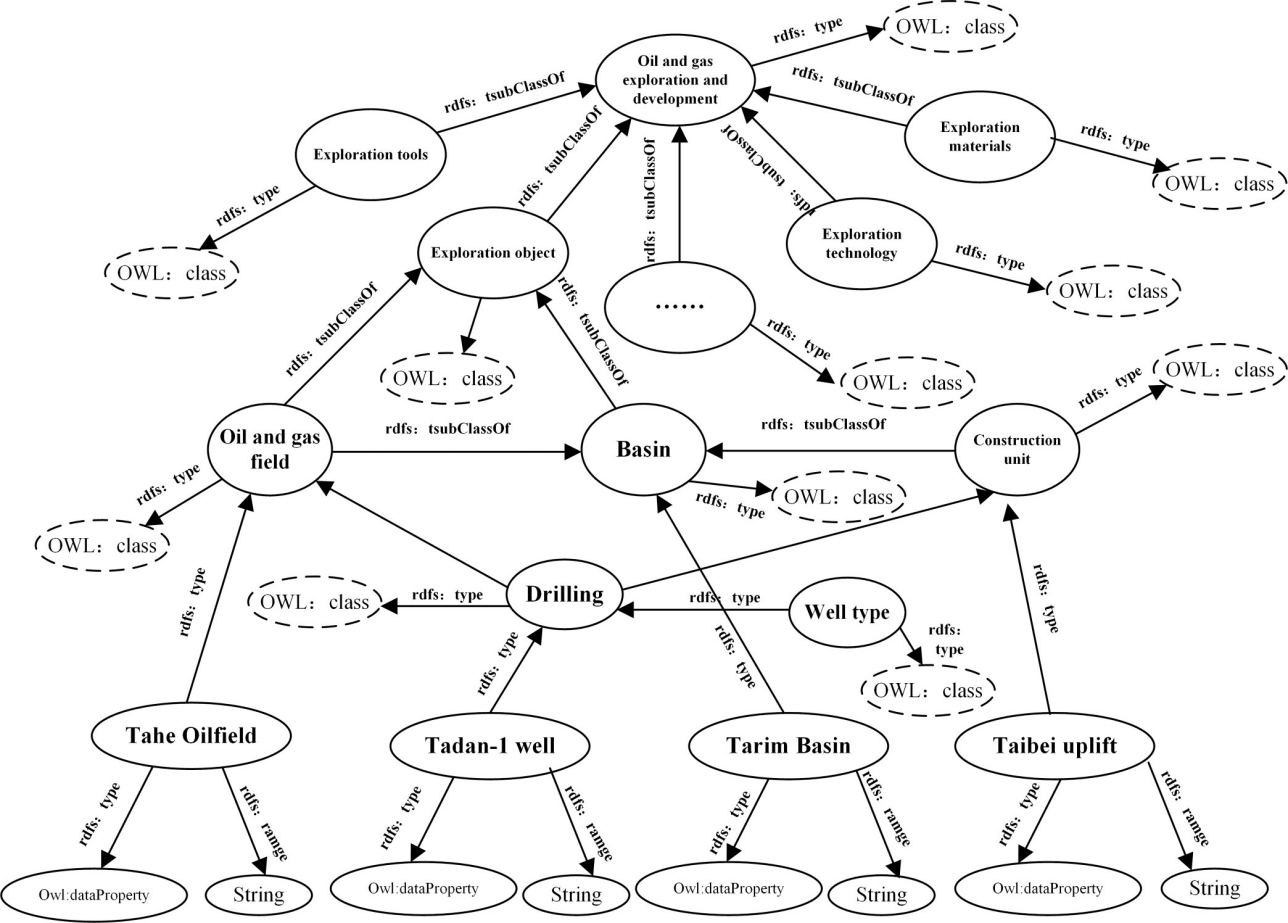
An example of an RDF directional marker map in the oilfield ontology.

The structure of oilfield ontology is a five-tuple O = {*C*,*R*,*Hc*,Re*l*,*Ao*} [18]. With the semantic basis of communication between different subjects, the ontology is composed of a specific set of terms that describe a particular situation in the oilfield, as well as a hypothetical set of corresponding representations. The ontology can not only describe the hierarchical structure of the concept, but can express other relationships between concepts, and constrain the connotative explanation of the concept by adding a set of appropriate relations, axioms, and rules. A complete ontology includes five elements: class (C), relation (R), attribute (At), axiom (Rel), and instance (Ao). Fig 4 is an example of an RDF directional marker map in the oilfield ontology. Among them, the solid-line ellipse represents the class, axiom, and instance of the ontology, the dashed ellipse represents the corresponding attribute, and the directed arrow represents the correspondence between the semantics.

1. Class (C). In addition to the general sense of the concept, the class can also indicate tasks, actions, events, and other names in the RDF triples expressed as the subject and object. For example, the triples (Oil and gas exploration and development, rdfs: type, OWL: class) is a class.
2. Relationship (R) describes the relationship between the concepts in the field by defining the constraints between the domain and the value domain. Among them, the definition domain is the concept in the concept collection, and the value domain may be the concept, data type, or numerical value composition. The main relationships between domain ontology concepts include subclass relations (subClassOf) and those between the instances and the concepts (edf: type). For example, subClassOf (geological object, oil and gas field) means “oil and gas fields” are subclasses of “geological objects”.
3. Attribute (At). The concept of domain ontology contains two attributes: the object attributes and the data attributes. The object attributes associate objects with each other, and the data attributes associate objects with data-type values. Concepts are also divided into four different relationships based on their interrelationships, as shown in Table 1, which classifies the relationships between concepts.
4. The axiom (Rel) is a description of the eternal truth, a reflection of reality.
5. Examples (Ao) are concrete examples of classes. In domain ontologies, instances inherit the attributes and relationships of related classes, and are the ranges in the domain ontology. For example, the Tadan-1 well (rdfs: type. OWL: Namedindividual) indicates that the well in the Tazhen-1 well is an example.

**Table 1:** Relationships between concepts.

Relationship Name	Relationship Description
Part-of	Represents the relationship between the concept of part and whole
Kind-of	Represents the inheritance relationship between concepts
Instance-of	Represents the concept of the relationship between examples and concepts
Attribute-of	Expresses another property of a concept

### 3.2 Neo4j storage features

A Neo4j graph is also known as the property graph (PG) [19]. *PG* = (*V*,*E*,*src*,*tgt*,*lbl*,∅) is a six-tuple representation of the label with a directed graph. Specifically, *V* is the set of nodes, *E* represents the set of edges, the function *src*:*E*→*V* indicates that each edge has a corresponding starting node, the function *tgt*:*E*→*V* indicates that each side has a corresponding termination node, the function *lbl*:*E*→*dom*(*S*) means that each edge has a label, and the function *ϕ*:*V*∪*E*→2^*P*^ represents a collection of property key-value pairs.

**Fig 5:**
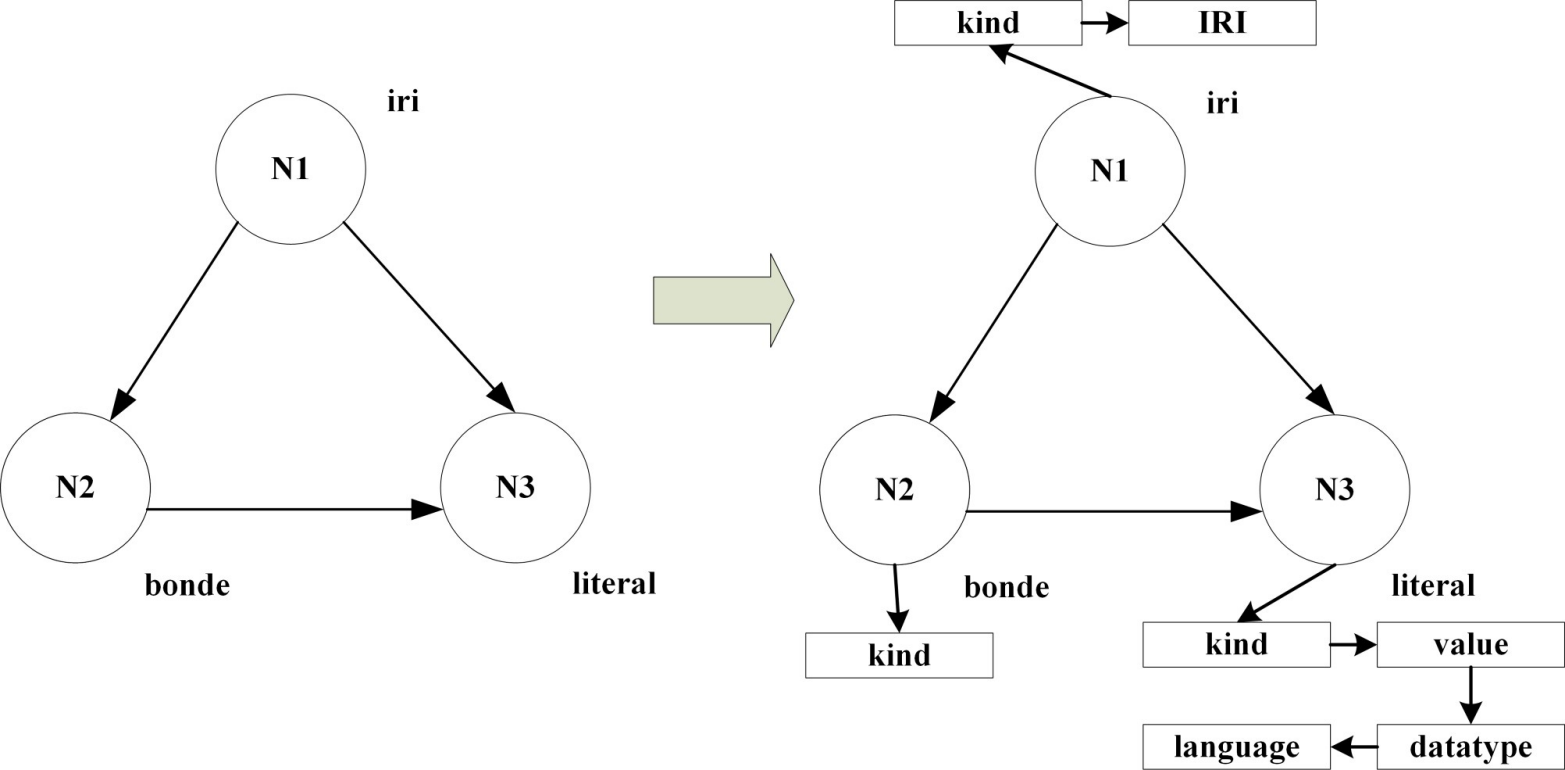
Neo4j database stored procedures.

Neo4j is a high-performance NOSQL graphics database whose basic structure is composed of nodes, relationships and attributes [20]. The nodes are designated as starting nodes and termination nodes, and two such nodes are connected by a relationship. The attributes are complementary to different nodes. Each node of Neo4j has a label, which can be divided into iri, literal, and bnode. The iri node has two attributes, kind and IRI, the bnode only has one kind of attribute, and the literal node has four attributes, which are kind, value, datatype, and language. The same node with different attributes is stored in the form of a linked list, as shown in Fig 5 for the Neo4j database stored procedure.

### 3.3 RDF data to Neo4j mapping rules

**Fig 6:**
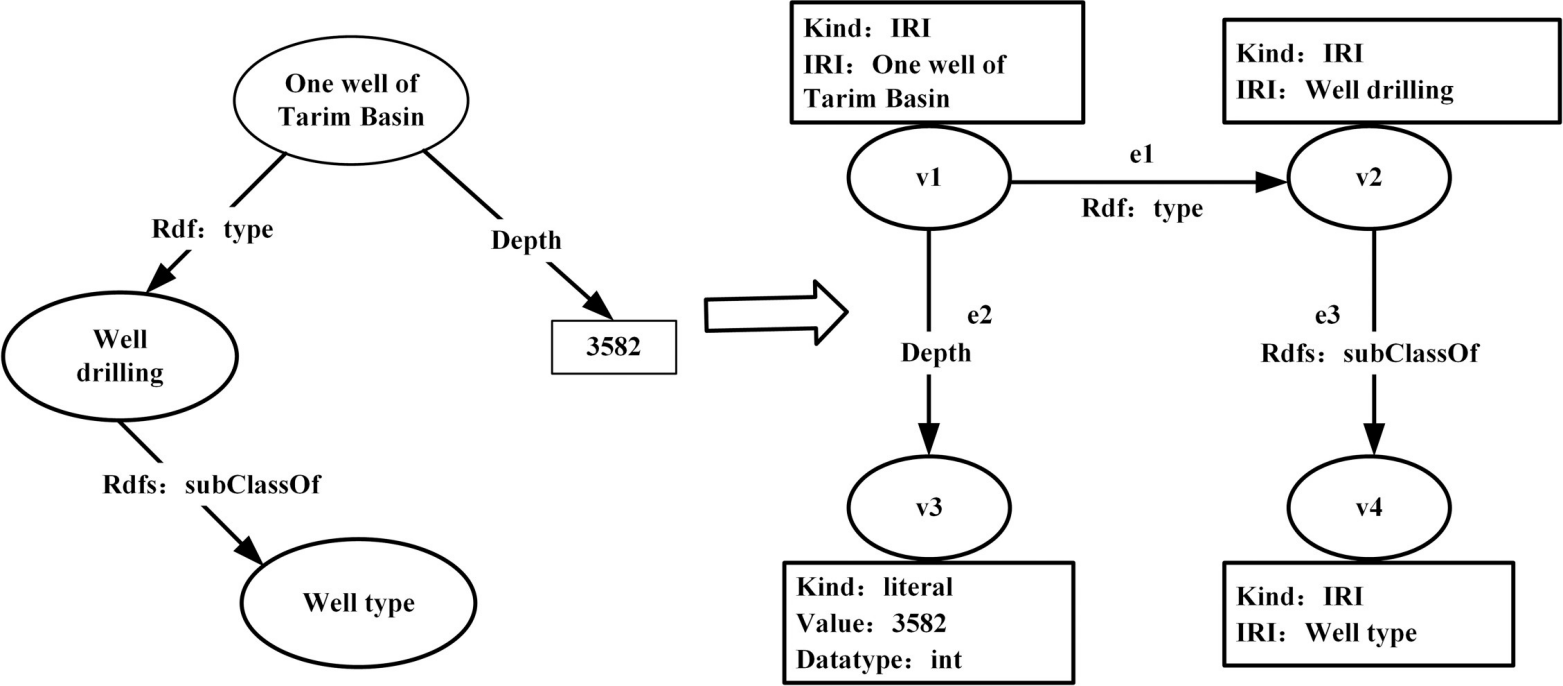
Mapping of RDF directed graph to Neo4j data structure.

The RDF directed graph is represented by the subject, predicate, and object, in which the subject can express the classes and the concepts. The object can not only represent the class and the concept, but can express the concept and the attribute of the class. The predicate expresses the relationship between the subject and object [21]. At the same time, the Neo4j data model and the RDF directed graph of the oilfield ontology information shown in Fig 4 have similarities in the structural model. We have implemented a series of mapping rules to map the RDF directed graph to the Neo4j data structure, as shown in Fig 6.

The following are the mapping steps from the RDF directed graph to the Neo4j database.

1. Traverse each attribute value in the RDF directed graph. In Neo4j, each attribute value in the RDF directed graph is generated by the corresponding node. Each node can establish multiple relationships with multiple nodes, and a single node can set multiple key-value pairs. For example, *V* = {*v*1,*v*2,*v*3,*v*4} is a set of nodes that are mapped in the RDF directed graph in the Neo4j database.
2. For each empty node (*bnode*)*v*(*b*) in the set *V* of nodes, the attribute set is obtained as *ϕ*(*v*(*b*)) = {〈"*kind*","*hash*"〉}, which means that the node has no extra attribute except the type label.
3. For each resource identifier node (*iri*)*v*(*u*) in the set *V* of nodes, we can obtain a collection of properties *ϕ*(*v*(*u*)) = {〈"*kind*","*IRI*"〉,〈"*IRI*","*im*(*u*)"〉}, which includes the node type and the attribute set of the “IRI” lable. For example,
  $$\begin{array}{c} \phi (v(u1))=\{\langle "kind","IRI"\rangle ,\langle "IRI","One\;well\;of\;tarin\;\mathrm{basin}"\rangle \}\\ \phi (v(u2))=\{\langle "kind","IRI"\rangle ,\langle "IRI","Well\;drilling"\rangle \}\\ \phi (v(u4))=\{\langle "kind","IRI"\rangle ,\langle "IRI","Well\;type"\rangle \} \end{array}$$
4. For each literal node (*literals*)*v*(*l*) in the set *V* of nodes, we can obtain an attribute set:
  $$\begin{array}{c} \phi (v(l))=\{\langle "kind","literal"\rangle ,\langle "value",vm^{-1}(l)\rangle \\ \langle "datatype",im(dtype(l))\rangle \}\cup lang. \end{array}$$
  We can get the “value”, “datatype” and “lang” attributes of the node type, where the “lang” attribute can be null. For example,
  ϕ(v(u3))={〈"kind","literal"〉,〈"literal",3582〉,〈"datatype",int〉}.
5. Each edge in the Neo4j database represents a different RDF triple. For example, *E* = {*e*1,*e*2,*e*3} is an edge set of the RDF directed graph map in the Neo4j database.
6. For each tuple *T* = 〈*s*,*p*,*o*〉, the labels of the edges correspond to *im*(*p*), and the starting and termination nodes are *v*(*s*) and *v*(*o*). For example,
  $$\begin{array}{l} src\left(e1\right)=v1,tgt\left(e1\right)=v2,lbl\left(e1\right)="rdf:type"\\ src\left(e2\right)=v1,tgt\left(e2\right)=v3,lbl\left(e1\right)="Depth"\\ src\left(e3\right)=v2,tgt\left(e3\right)=v4,lbl\left(e3\right)="rdfs:subClassOf" \end{array}$$

This paper is mainly based on the Java Jena API method in the Eclipse environment to achieve the RDF file to Neo4j map data storage model conversion.

### 3.4 Retrieval method based on a two-tier index architecture

The SPARQL protocol and the RPF query language are one of the core technologies of Semantic Web. The SPARQL protocol is a protocol for RDF to develop ontology-retrieval language and data collection, the RPF query language is used to access and manipulate RDF data [22]. It is one of the core technologies of the Semantic Web. When RDF data must be associated with the retrieval, it is necessary to construct the SPARQL query and perform multiple query tasks, which are not conducive to our query request. Combining with the characteristics of multidisciplinary fields in the oilfield, we propose a retrieval algorithm for the ontology of petroleum domain that achieve the ontology query function by using CYPHER [23] search language and Apache Solr [24] indexing technology.

First, we need to create a two-tier index architecture, including an object indexing mechanism and a triple indexing mechanism. The first layer is used for object indexing and the second layer is used for triple indexing. Tables 2 and 3 respectively show object and triad index. Among them, the object index is to arrange the information with retrieval meaning in an orderly manner to establish an object index table, which achieve the purpose of rapid classification and retrieval. The information having the meaning of retrieval may be a unique identification id, an object name, and the like. The object index table consists of object attributes and corresponding label descriptions. The object properties are used to describe the nature of an object, including numbering, name, alias, and entity type, which is a feature that distinguishes an object from other objects. All objects in Table 2 are assigned an id number by the indexing mechanism to build an index table, which is convenient for querying objects quickly.

**Table 2:** Object index.

Attribute	Description
Numbering	id
Name	label
Alias	altLabel
Entity type	entityType

**Table 3:** Triad index.

Attribute	Subject		Predicate	Object
Numbering		sID	pID	oID
Name		sLabel	pLabel	oLabel
Type		sType	pType	oType
Type name		sTypeValue	pTypeValue	oTypeValue

The triplet index is an index method formed by taking the subject resource, the predicate, and the object resource as an object, which is a specific representation of the object index. The predicates are used to describe and determine the relationship between object properties, features, or objects. In Table 3, the subject, the predicate, and the object are all special objects. The object is uniquely identified by the id, which embodies the semantic information between various concepts.

When the search engine analyzes the search statements for the user, we use a two-tier index architecture to construct the retrieval expressions according to the retrieval requirements, and performs the retrieval tasks.

**Fig 7:**
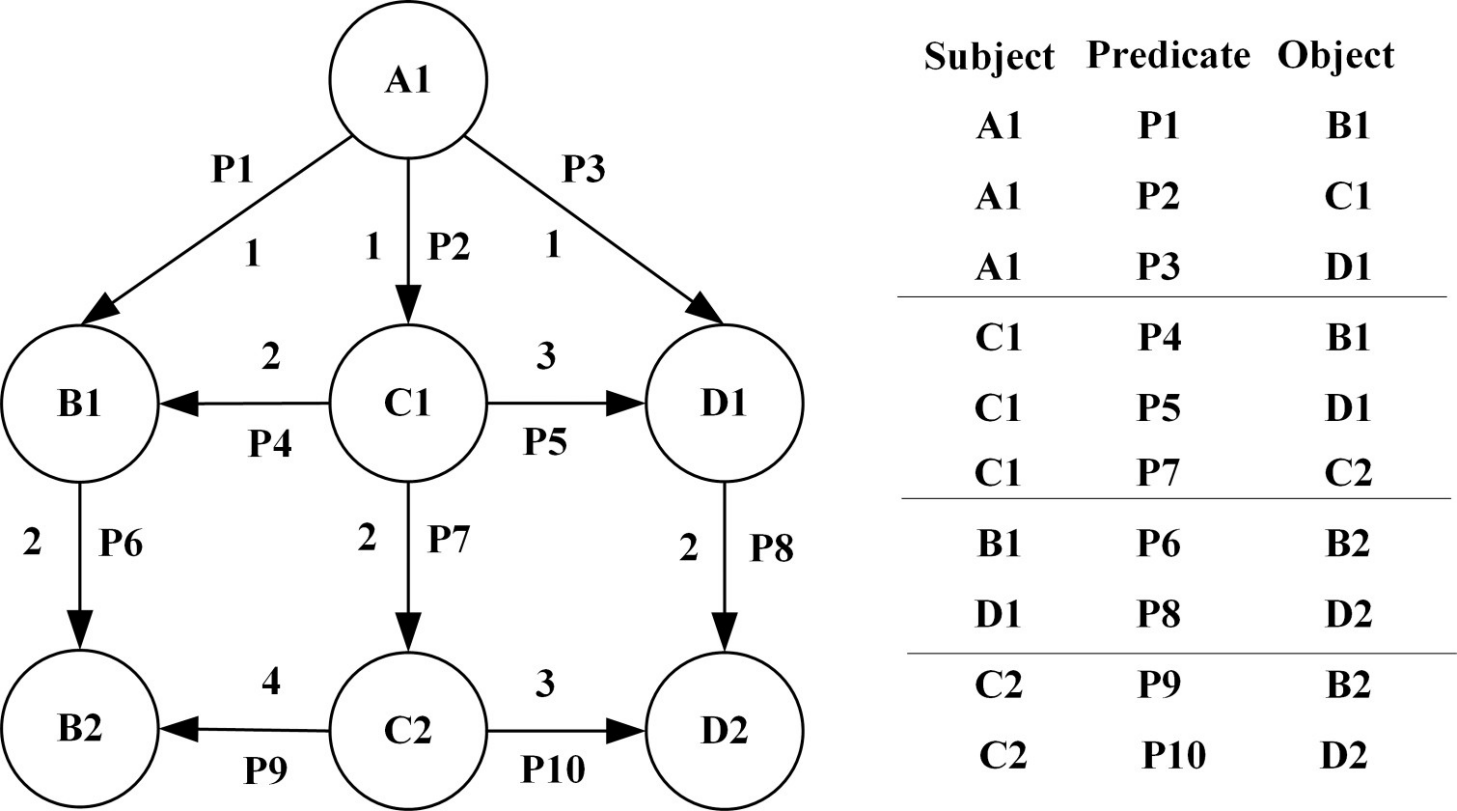
Graphic structure of multiple sets of triples in relational degree retrieval.

1. Match object retrieval: Object matching retrieves the object index by querying the result of exact matching or fuzzy matching, and sorting the result set to obtain the correlation of the result set. For example, the exact matching of the object named "Tarim Basin" is retrieved, and its search is
  Query=(label:"TarimBasin")or(altLabel:"TarimBasin")
2. Relationship matching search: This is mainly used for a triad index of a retrieval method. In the triple (*s*,*p*,*o*), we must retrieve the relationship between *s* and *o*. For example, when we search for the “exploration technology” object, the search engine first finds the ID corresponding to the “exploration technology” in the ID index table, and obtains the corresponding ID of “9672”. Then the search for the structural relationship is:
  Query=(sID:"9672")or(oID:"9672")
  If we know the familiarity of the object and its designation, we can retrieve another corresponding object by using the *s* and *p* or *o* and *p* known by the triple, and we need to use the object *o* or *s* to retrieve another match. For example, we need to search for the components of “petroleum”, and combine mutual rule systems. The given search structure is:
  $$\begin{array}{c} Query=\left(sLabel:"oil"\right)\;and\;\left(pLabel:"composition"\right)\\ or\;\left(pLabel:"composition"\right)\;and\;\left(sLabel:"oil"\right) \end{array}$$
3. Relational degree retrieval: This is a retrieval method defined according to the graphical structural features formed by multiple sets of triples in the oilfield ontology, and each triplet represents a relationship, as shown in Fig 7. The left graph shows the graphical structure formed by multiple sets of triples, and the right graph shows the relationship details of multiple triples. When two objects are known to be different, such as *B*2 and *D*2, we define the path by querying the degree of relationship weight between the instance nodes *A*1 corresponding to the two objects. The degree of relationship weight is determined by the association and similarity of two objects in knowledge engineering. If the degree of similarity is larger, the relationship weight is smaller, then the distance between the two nodes is closer in the figure. These paths are defined as options such as “near”, “far”, “closer” and “further” depending on the size of the relationship weight, which is the distance factor in the graph.

According to different semantic relations, we may have different results for the relational degree retrieval of the same two objects. For example, we query the relational degree between *B*2 and *D*2.When querying along the path *A*1→*B*1→*B*2 and *A*1→*D*1→*D*2, the length of the paths are 6, and we define both as “closer”. However, when queried along the path *A*1→*C*1→*C*2→*B*2 and *A*1→*C*1→*C*2→*D*2, the length of the path is 7 and 6, respectively, and we define both as “near”. Finally, we return the path from node to root in different semantics by means of map traversal, providing more path selection for relational retrieval. The Neo4j database's relational degree retrieval and map traversal mechanism provides flexible access to query results through CYPHER queries. For example, we need to query the relationship between “Shengli Oilfield” and “Tarim Oilfield”. Then the CYPHER query statement is:

$$\begin{array}{c} start\;a=node\left(*\right),b=node\left(*\right)match\;p=a-\left[*0.2\right]-b\\ where\;was\;a.label="Shengli\;Oilfield"\;and\;b.label="Tarim\;Oilfield"\\ return\;p\;order\;by\;length\left(p\right) \end{array}$$

## Experiment

### 4.1 Experimental design

The purpose of our evaluation was to: (1) see the query response time for datasets of different sizes; (2) see the storage scale for datasets of different sizes; (3) compare our methods with traditional methods by loading a full dump into a relational database and extracting by query

### 4.2 Experimental setup

To further validate the advantages of this method, we carried out a test with the Berlin SPARQL Benchmark (BSBM) [25] standard dataset. We divided the dataset into five different datasets by size and named them a, b, c, d, and e, as shown in Table 4. We used these five data sets to test and verified that when the size of the data set is growing, our proposed method is significantly better than the traditional relational database in terms of space usage and retrieval time. In addition, using the same size data set, we added the method of triple index direct index storage as an experimental comparison to get the results of data storage and retrieval. We measured the performance of our approach in terms of runtime and storage consumption. All experiments were implemented on a Windows 10 machine with an Intel Core i5-8400 processor, 8 GB of RAM, and a 120-GB SSD.

**Table 4:** Sizes of five groups.

Numbering	A	B	C	D	E
RDF triples Size (unit)	50,000	250,000	1,000,000	5,000,000	25,000,000

### 4.3 Implementation

In the RDF data-storage experiment, first, we store the RDF data in a triplet manner, and name it Ta, Tb, Tc, Td, and Te on five different data sets. Then we put the RDF data triples into the Neo4j database, stored them in Java, and manipulated them using our datasets with different sizes, naming them Na, Nb, Nc, Nd, and Ne. At the same time, datasets of the same size are stored in a relational database by creating tables and other forms. We name them Ra, Rb, Rc, Rd, Re.

In the search-efficiency comparison experiment, we use the triple direct index method to retrieve data for Ta, Tb, Tc, Td and Te, and record the corresponding time. By using the retrieval method based on the two-layer index architecture, we searched Na, Nb, Nc, Nd, and Ne, and recorded the time consumed by the Cypher language retrieval method proposed in this paper. We similarly searched Ra, Rb, Rc, Rd, and Re using the traditional SQL query language, and recorded the retrieval time. We compared the experimental results of the five dataset sizes on query efficiency under the same method, and compared our proposed storage method with the traditional method with the same dataset sizes.

### 4.4 Results

**Fig 8:**
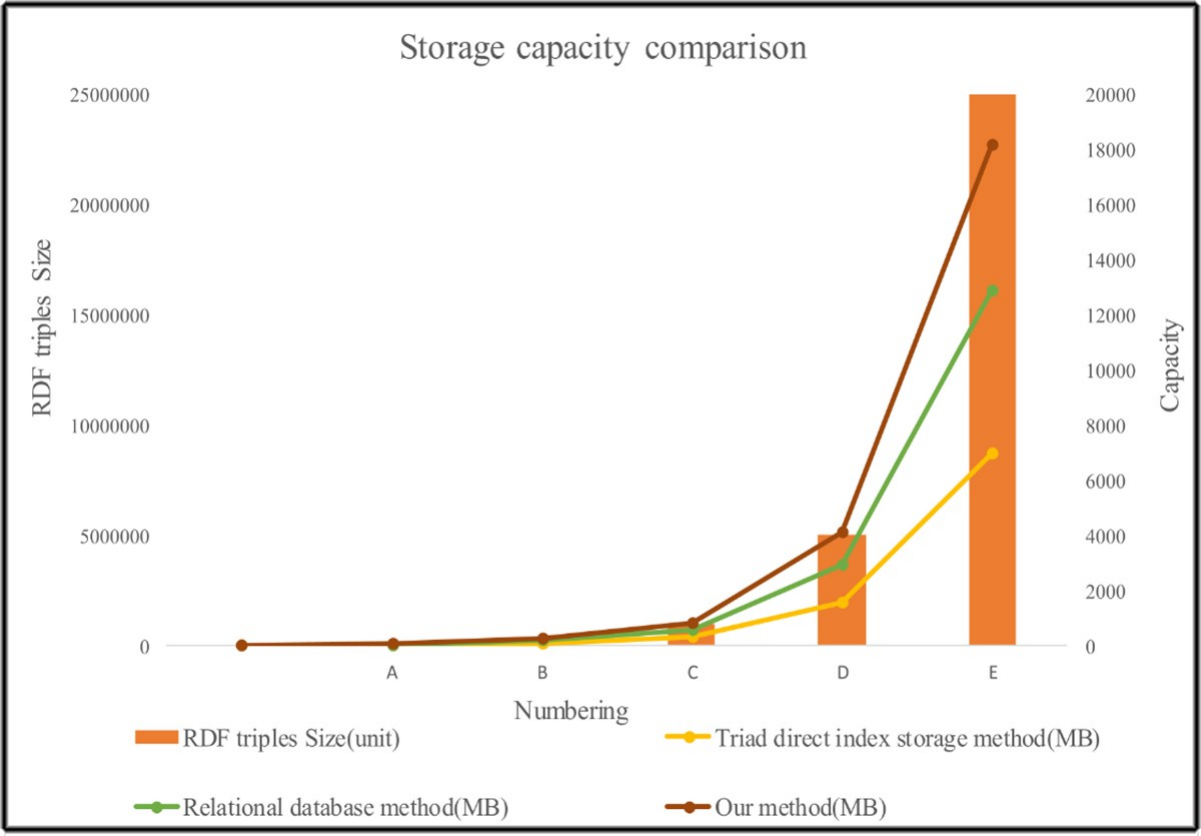
Storage capacity comparison chart.

**Fig 9:**
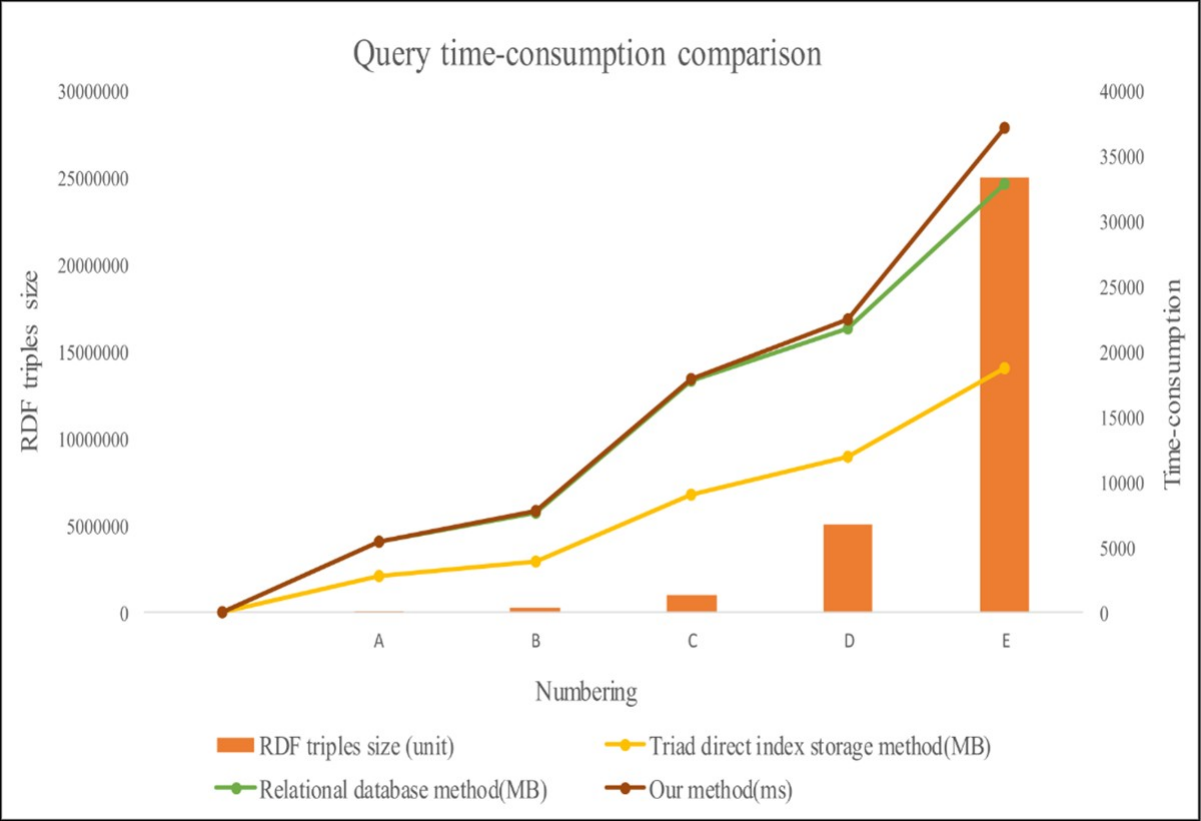
Query time-consumption comparison chart.

In Figs 8 and 9, the brown line represents triad direct index storage method, the green line represents the storage method of the traditional relational database, and the yellow line represents our storage method. These compare the size of the storage space and the retrieval time of information from datasets of different sizes. We can draw the following conclusions.

1. In the case of using the same dataset size, our storage method can save 30.8% of the storage space compared to the simple use of the triad direct index storage method. At the same time, if we use traditional relational database for storage, our method is also superior, which can save 13.04% (S1 Table) storage space.

2. With the same dataset size, our search method is 30 (S2 Table) times more efficient than the traditional SQL query method. At the same time, the retrieval efficiency of the traditional relational database SQL query method is slightly better than the triple index direct method.

3. With increasing dataset sizes, the proposed storage mapping and retrieval methods are more adaptable than traditional methods.

## Conclusions

In this paper, we propose a domain ontology building process based on the Neo4j graphics database and a retrieval method based on a two-tier index architecture. Our assessment shows that our approach can save 13.04% of the storage space and is 30 times more efficient compared to relational databases. These methods are the main steps toward building large-scale domain ontology. Some neural-like models should be involved into the dataset, like spiking neural networks and artificial intelligent neural networks [26–31], for further research in processing the data. Also, some chemical data and methods can be considered as functional modules in Neo4j graph database [32–36].

As the first step of a larger research agenda, this work dramatically improves large-scale RDF data management. The proposed approach focuses only on two of five stages of the process of building the domain ontology. In the future, we aim to support the subsequent stages of ontology expansion and multi-ontology integration. We envision that organizations will be empowered to seamlessly integrate RDF data into an existing ontology. A particular challenge is the mapping of RDF data to existing internal information structures and the establishment of a co-evolution between private and public data involving continuous update propagation from RDF sources while preserving revisions applied to prior versions of these datasets.

## Supporting information

S1 Table Store experimental result data. (XLSX)

S2 Table Query experimental result data. (XLSX)
